# Trends in Diabetes Mortality in Urban and Rural China, 1987–2019: A Joinpoint Regression Analysis

**DOI:** 10.3389/fendo.2021.777654

**Published:** 2022-01-17

**Authors:** Binbin Su, Yiran Wang, Yanhui Dong, Gang Hu, Yike Xu, Xiaobo Peng, Qianyun Wang, Xiaoying Zheng

**Affiliations:** ^1^ Institute of Population Research, Peking University/PKU-APEC Health Science Academy, Beijing, China; ^2^ Institute of Child and Adolescent Health, School of Public Health, Peking University Health Science Center, Beijing, China; ^3^ Gabelli School of Business, Fordham University, New York, United States; ^4^ Department of Nutrition and Food Hygiene, Hubei Key Laboratory of Food Nutrition and Safety, Tongji Medical College, Huazhong University of Science & Technology, Wuhan, China

**Keywords:** diabetes mellitus, mortality, trends, joinpoint regression analysis, annual percentage change

## Abstract

**Purpose:**

Diabetes mellitus is emerging as an epidemic worldwide, and the incidence and prevalence of diabetes have drastically changed in China over the past 30 years, but data on its mortality rate are scarce. This study aimed to analyze the time trends of mortality rates among patients with diabetes in the rural and urban population in China between 1987 and 2019.

**Methods:**

The research data come from China’s annual report on national health statistics and the Chinese Health Statistics Yearbook. Age-standardized mortality rates were calculated by using the direct method based on the World Standard Population from the WHO. Joinpoint regression analysis was employed to estimate the annual percent change and average annual percentage changes of mortality rates of diabetes mellitus.

**Results:**

An overall trend for increment in diabetes mortality was observed. The crude mortality rates and age-standardized mortality rates of diabetes for urban and rural residents in China showed a significant increasing trend between 1987 and 2019. Mortality due to diabetes in urban areas has been higher than in rural areas for 30 years. However, due to the rapid increase of rural diabetes mortality in the past decade, the gap between the two gradually narrowed. The age-standardized mortality rates of diabetes increased by about 38.5% in urban areas and 254.9% in rural areas over the whole study period. In addition, the age-standardized mortality rate of females with diabetes was higher than that of males, but this pattern began to change in urban areas in 2012. Finally, the age-standardized mortality rates in the elderly population in China are higher with a faster growth rate, especially in rural areas.

**Conclusion:**

The mortality rate of diabetes is on the rise in China. The rapid growth of the mortality rate of diabetes in rural areas leads to the reduction of the urban–rural gap. Male mortality rates in urban areas have surpassed those of women. At the same time, the mortality rate of diabetes showed obvious elder-group orientation. As China’s population ages, the burden of death and disability caused by diabetes and its complications will continue to increase. These results indicate that diabetes has become a significant public health problem in China. Such an effect increases the demand for strategies aimed at the prevention and treatment of diabetes mellitus. In addition to the prevention and intervention of diabetes in high-risk groups, it is also necessary to establish diabetes screening networks to identify patients with mild symptoms. Early detection and timely intervention can effectively reduce the incidence and mortality of diabetes.

## Research in context

-What is already known about this topic?

Diabetes mellitus is becoming a global public health problem. The prevalence of diabetes in China is particularly severe. In recent years, the prevalence and mortality rates of diabetes in China are rising rapidly, and the secular trends in mortality remain unknown.

-What is added by this report?

This study analyzed the chronological trend of diabetes mortality in China’s urban and rural areas, as well as the mortality rate in different age groups and gender groups. This is helpful for us to understand the trend of diabetes in China and provide valuable scientific evidence for further improvement of China’s diabetes prevention strategies.

-What are the implications for public health practice?

The results of this study showed that the mortality rate of diabetes mellitus in China has increased considerably in the last several decades and varies among different areas and population groups, highlighting the importance of region-specific and group-specific preventions and treatments. Early screening and timely treatment of diabetes are necessary, especially in rural China, while the prevention and treatment of diabetes among the elderly population need to be urgently introduced.

## Introduction

Diabetes mellitus (DM) is a severe chronic disease that occurs either when the pancreas does not produce enough insulin (a hormone that regulates blood glucose) or when the body cannot effectively use the insulin it produces (1). Persistent hyperglycemia and long-term metabolic disorder may lead to systemic organ damage, dysfunction, and failure (2). DM is also a known risk factor for blindness, vascular brain diseases, renal failure, and limb amputations (3). Diabetes of all types can lead to complications in many parts of the human body and increase the overall risk of premature demises. DM is emerging as an epidemic all over the world (4). In 2019, the International Diabetes Federation (IDF) reported that 463 million adults worldwide suffer from diabetes. If effective preventive measures are not taken, it is expected to increase to 700 million by 2045 (5). According to the latest data released by IDF, about 6.7 million adults will die of diabetes and its complications in 2021, equivalent to one death every 5 s.^1^

The epidemic of diabetes is one of the most alarming public health issues of the 21st century, especially for lower-middle-income countries (6). Compared with high-income countries, the prevalence of diabetes in middle-income and low-income countries develops faster (7). It was predicted that from 2010 to 2030, there will be a 67% increase in the prevalence of diabetes in these countries (8). Although from a global perspective, the overall health level has improved and life expectancy has increased, diabetes is still the second largest factor affecting life expectancy (9). There was also an increase in both type 1 and type 2 diabetes prevalence among children and adolescents (10). As the largest developing country globally, China currently has an enormous number of diabetic patients. With a large population, China has 35.5 million people who are 65 years of age or older, and it is expected to increase to 54.3 million by 2030 (5). With the deepening of aging in China, the disease burden and problems caused by DM will be further expanded.

The primary purpose of this study was to examine the trends of temporal changes in the annual motility rate of DM in China during 1978–2019, using the joinpoint regression model developed by Kim et al. (11), which can be used to deal with long-term disease data of multiple trend segments scientifically and fit well. We also compared the disparity of mortality rates between the urban and rural areas, which would be useful for preventing and controlling DM in the future.

## Methods

### Data Sources

The DM cases were defined by the International Classification of Diseases (ICD) codes: ICD-9 (249–250) for data collected before 2001 and ICD-10 (E10–E14) for data from 2001 onwards. The mortality data in urban and rural populations were derived from the Chinese Health Statistical Annual Report (1988–2002), which are not published publicly, China Health Statistics Yearbook (2003–2012), China Health and Family Planning Statistics Yearbook (2013–2016), and China Health Statistics Yearbook (2017–2020). The mortality statistics were obtained by the Ministry of Health-Vital Registration System. The demographic data for urban and rural residents were derived from China Population Statistics Yearbook (1991–2006) and China Population and Employment Statistics Yearbook (2007–2020) collected by sample survey and census. Data were available by gender and age groups, from 0, 1–4, 5–9 to 80–84 and up to 85+ years. The trends in age-specific mortality of DM before 15 years old were not analyzed owing to the extremely low mortality.

### Trends in Mortality Rates

Mortality and population data were organized into 5-year age groups, up to 85+ years, to correspond with age categories used in the WHO World Standard Population (WSP) as reference (12).

We calculated age-standardized mortality rates (ASMRs) per 100,000 with 95% CI for each study year (1987–2019) using the direct method, based on the WSP and annual age-specific crude mortality rates (CMRs). To calculate the annual variation in mortality rates and identify significant change points, we performed joinpoint regression analysis using Joinpoint Regression Program from the Surveillance Research Program of the National Cancer Institute Version 4.9.0.0 (Statistical Research and Applications Branch National Cancer Institute, USA).

Joinpoint analysis identifies the best fit for inflection points (“joinpoints”) at which there is a significant change in trends using a series of permutation tests, with Bonferroni adjustment for multiple comparisons (13). In this study, joinpoint analysis was used to identify years (as the independent variable) with significant changes in mortality rate over the study period and the size of these changes (as the percentage change in rate per year). Using a natural log-linear model enables the analysis of a constant percentage change in rate over time. We allowed up to five joinpoints by utilizing a Monte Carlo permutation method and evaluated whether there was a difference from no shift in each segment using z-test and a p-value of less than 0.05 as statistically significant.

## Results

### Trend of Crude Mortality and Age-Standardized Mortality of Diabetes Mellitus in Urban and Rural Areas of China

**Figure 1:**
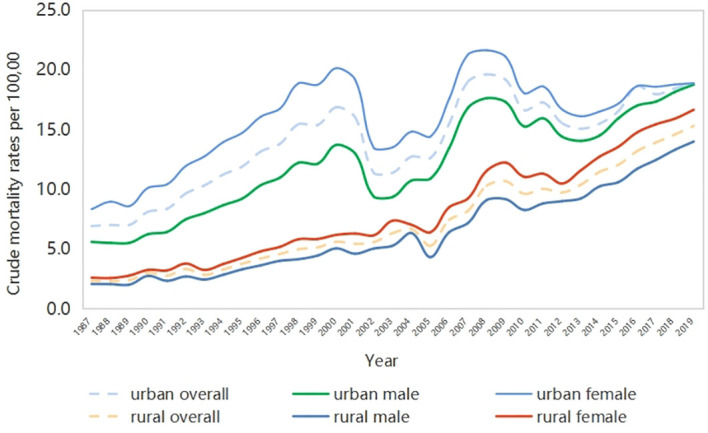
Trend of crude mortality rates of diabetes in China 1987–2019.

As shown in **Figure 1**, the CMRs of DM (DM-CMRs) in urban and rural areas of China show an overall upward trend during the period of interest, but there are certain fluctuations in urban mortality rates. The DM-CMR in urban areas is higher than that in rural areas, and that in females is higher than in males. From the perspective of gender differences, the gap of DM-CMR in urban areas has been decreasing in recent years, while that in rural areas is expanding.

**Figure 2:**
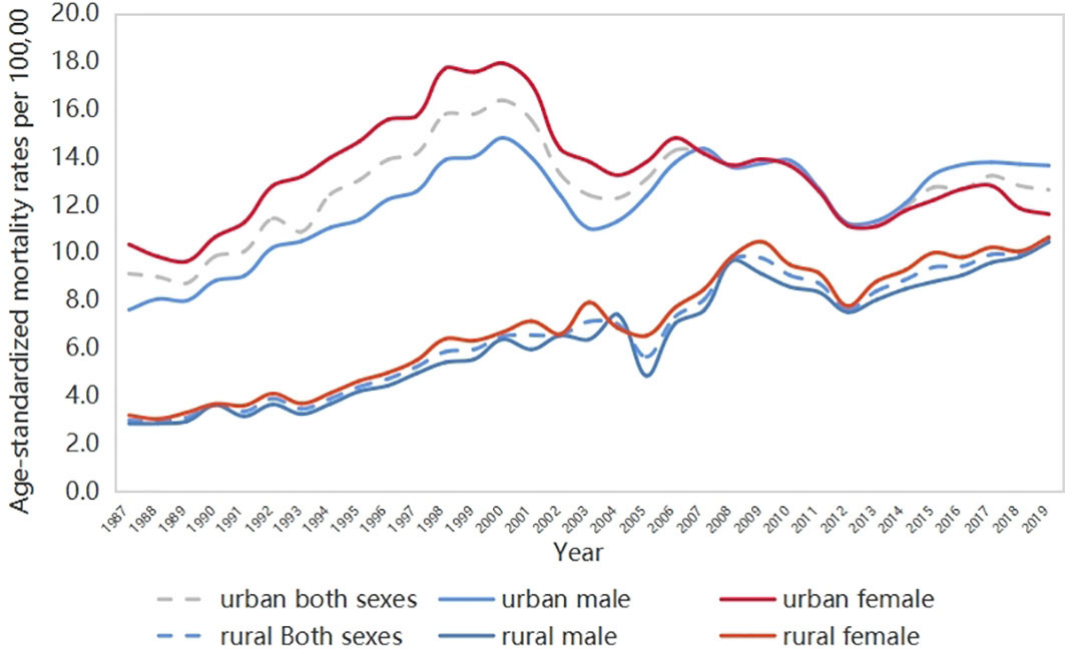
Trend of age-standardized mortality rates of diabetes in China 1987–2019.

The ASMRs of DM (DM-ASMRs) from each year in urban males and females, and rural males and females between 1987 and 2019 are shown in **Figure 2**. Continuing increasing trends on ASMR were observed in both rural males and females, while fluctuations occurred in urban areas. The ASMR of DM in the urban population is generally higher than that in the rural population during the whole study period, but the gap is gradually narrowing. Most of the time, the ASMR of females in urban areas was higher than that in males, but this situation changed after 2012. This indicates that the mortality rate of DM among men in urban areas of China has increased rapidly in the past decade. At the same time, the gender gap of ASMR in rural areas has become smaller.

The CMR and ASMR of DM at the beginning (1987) and the end (2019) of the study period are shown in **Table 1**, followed by the average annual percentage changes (AAPCs) during the 33 years and annual percentage change (APC) for each sub-period.

**Table 1:** Joinpoint analysis of crude and age-standardized mortality rates of diabetes in urban and rural areas.

	Mortality rate (per 100,000)		Total study period^b^		Period 1		Period 2		Period 3		Period 4		Period 5		Period 6	
	1987	2019	AAPC (%)	95% CI	Years	APC (%)	Years	APC (%)	Years	APC (%)	Years	APC (%)	Years	APC (%)	Years	APC (%)
**Crude mortality**																
**Diabetes in urban areas**																
**Both sexes**	87.0	16.6	3.5*	1.8, 5.2	1987–2000	7.82*	2000–2003	−14.51*	2003−2008	12.91*	2008−2013	−5.32	2013−2019	4.32*	–	
**Male**	6.4	15.9	4.3*	2.6, 6.0	1987–2000	7.97*	2000–2003	−13.15	2003−2008	14.76*	2008−2013	−4.73*	2013−2019	5.35*	–	
**Female**	10.4	17.2	2.8*	1.2, 4.4	1987–2000	7.58*	2000–2003	−15.28*	2003−2008	11.43*	2008−2013	−6.01	2013−2019	3.26*	–	
**Diabetes in rural areas**																
**Both sexes**	2.8	12.1	6.4*	4.1, 8.7	1987–2000	7.43*	2000–2005	1.82	2005–2008	19.40	2008–2012	−1.01	2012–2019	6.86*	–	
**Male**	2.3	10.6	6.4*	6.0, 6.8	–										–	
**Female**	3.2	13.6	6.3*	4.1, 8.5	1987–2000	7.47*	2000–2005	1.92	2005–2008	19.22*	2008–2015	1.50	2012–2019	6.64*	–	
**Age-standardized mortality****^a^**																
**Diabetes in urban areas**																
**Both sexes**	10.0	12.7	1.4	−1.0, 3.9	1987–2000	5.40*	2000–2003	−9.31	2003–2009	2.61	2009–2012	−7.41	2012–2019	2.01	–	
**Male**	9.0	13.3	1.9*	0.4, 3.4	1987–2000	5.50*	2000–2003	−10.21*	2003–2007	7.52*	2007–2013	4.01*	2013–2016	6.61	2016–2019	−0.51
**Female**	11.3	12.2	0.8	−1.1, 2.8	1987–2000	5.40*	2000–2003	−10.21	2003–2009	0.91	2009–2012	−6.92	2012–2019	0.92	–	
**Diabetes in rural areas**																
**Both sexes**	3.4	9.4	4.3*	1.9, 6.8	1987–2002	6.40*	2002–2005	−4.46	2005–2008	17.41	2008–2012	−4.89	2012–2019	3.94*	–	
**Male**	3.2	8.8	4.4*	1.6, 7.2	1987–2002	6.35*	2002–2005	−5.11	2005–2008	18.93	2008–2012	−5.22	2012–2019	4.35*	–	
**Female**	3.6	10.0	4.0*	2.1, 5.9	1987–1993	3.89*	1993–2000	8.73*	2000–2005	−0.36	2005–2009	11.19*	2009–2012	−7.51	2012–2019	3.83*

AAPC, average annual percent change; APC, annual percent change.

*The AAPC/APC is significantly different from 0 at the alpha = 0.05 level.

a Standardized to the WHO standard population.

b Years 1987 to 2019.

- No joinpoints identified.

The joinpoint analysis indicated that from 1987 to 2019, the CMR of DM in both urban areas and rural areas in China showed a significant growth trend, with an average annual growth rate of 3.5% (95% CI 1.8–5.20) and 6.4% (95% CI 4.1–8.7), respectively. The yearly mortality of diabetes significantly increased since 1987 by +4.3% (95% CI 2.6–6.0) in the urban male population while +2.8% (95% CI 1.2–4.4) in urban females. Meanwhile, the average annual growth rate of DM mortality in males and females in rural China is about 6.4% (95% CI 4.1–8.7), much higher than that in urban areas.

The ASMR of diabetes significantly increased since 1987 by +4.3% (95% CI 1.9–6.8) yearly in rural areas, and two joinpoints were identified for the rural population (a significant increase by 6.40% (p < 0.05) from 1997 to 2002 and an insignificant decrease from 2002 to 2012, followed by a considerable increase by 3.94% (p < 0.05) from 2012 onwards). More in-depth analyses of the chronological trends in men revealed that there are two turning points in the trend of mortality from DM, which appeared in 1987–2002 and 2012–2019.

For women, four joinpoints were detected during the whole study period. There was a significant increase in mortality between 1987 and 1993, reflected in the APC of +3.89% (p < 0.05). From 1993, the mortality rate increased until 2000, with a significant APC of +8.73% (p < 0.05). Two turning points appeared in 2005–2009 and 2012–2019. The rate did not significantly change between 2000 and 2005 (APC = –0.36%; p > 0.05) but increased sharply between 2005 and 2009 (APC = –7.2%, p < 0.05) and was then followed by a significant increase of 3.83% (p < 0.05) from 2012 to 2019.

### Trends in Age-Specific Mortality Rate

Across the entire period, the DM-ASMR for adults aged 40 to 45 years showed no significant change in rural and urban China but varied among other age groups.

In urban population, the DM-ASMR increased significantly in the age groups 50–55 and 75+ during the whole study period but decreased significantly in the age group 20–25, with −2.9% (95% CI −4.0, −4.7) per year. In terms of gender, the rate for female decreased significantly in the age groups 20–25, 25–30, 30–35, and 55–60 but increased significantly in the age groups 75–80, 80–85, and 85+. The corresponding APC values are 1.0% (95% CI 0.2, 1.8), 2.4% (95% CI 1.8, 3.0), and 6.1% (95% CI 3.1, 9.3), respectively. The rate for male increased significantly in the age groups 55–60, 70–75, 75–80, 80–85, and 85+. The corresponding APC values are 2.2% (95% CI 0.7, 3.6), 1.0% (95% CI 0.2, 1.7), 1.0% (95% CI 0.2, 1.7), 1.7% (95% CI 1.1, 2.4), and 3.6% (95% CI 3.0, 4.3), respectively.

In rural populations, the DM-ASMR of DM has shown a significant growth trend in those aged 55 years or older, which is much higher than that in urban areas. There was no significant change of ASMR in the age group under 55 years. In terms of specific age groups, the ASMR increased by +3.8% (95% CI 1.9, 6.8) yearly in the age group 60–65, +3.4% (95% CI 2.5, 4.4) yearly in the age group 65–70, +4.5% (95% CI 3.4, 5.6) and +5.0% (95% CI 3.9, 6.1) yearly in the age group 70–75, +5.1% (95% CI 4.2, 6.0) yearly in the age group 75–80, +6.4% (95% CI 4.9, 8.0) yearly in the age group 80–85, and +7.9% (95% CI 6.9, 9.0) yearly in the age group 85+.

**Figure 3:**
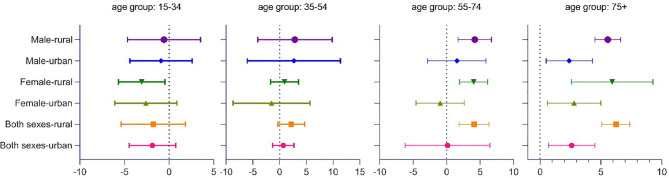
Average change in age-standardized mortality rate of DM per year. Years 1987 to 2019. Error bars as 95% CIs. DM, diabetes mellitus.

Further analysis found that the DM-ASMR for females decreased in the age groups 30–35 and 35–40, with −3.2% (95% CI −4.8, −1.5) and −2.9% (95% CI −3.9, −1.9) per year, respectively. However, in those aged 55 years or older, the ASMR of DM has shown a significant increasing trend. The ASMR of urban males increased by +3.6% (95% CI 2.5, 4.6) yearly in the age group 60–65, +4.5% (95% CI 3.1, 6.0) yearly in the age group 65–70, +4.8% (95% CI 3.5, 6.0) yearly in the age group 70–75, +4.5% (95% CI 3.5, 5.5) yearly in the age group 75–80, +5.4% (95% CI 4.0, 6.7) yearly in the age group 80–85, and +5.6% (95% CI 5.3, 7.8) yearly in the age group 85+. The pattern of DM mortality trend for women in rural areas is similar to that of men. However, the significant increase in mortality rate occurred earlier and increased faster (**Table 2**). As shown in **Figure 3**, from the study period, the elderly group (75+) showed a significant increase in DM-ASMR, and in rural areas, this situation expanded to as early as 55 years old. This indicates that the mortality rate of diabetes in the Chinese elder population has been increasing over the past several decades, and the situation in rural areas is severer.

**Table 2:** Annual percent changes (APCs) in age-specific mortality rates of diabetes in urban and rural areas.

Age group	Diabetes in urban areas						Diabetes in rural areas					
	Both sexes		Male		Female		Both sexes		Male		Female	
	Average APC (%)	95% CI	Average APC (%)	95% CI	Average APC (%)	95% CI	Average APC (%)	95% CI	Average APC (%)	95% CI	Average APC (%)	95% CI
15–	−0.9	−3.8, 2.1	1.0	−7.2, 9.8	−5.5	−15.2, 5.2	−2.1	−7.9, 4.1	−6.8	−17.0, 4.6	−5.5	−13.4, 3.1
20–	−2.9	−4.0, −1.7	−1.8	−4.0, 0.4	−3.4	−4.4, −2.5	−2.1	−5.2, 1.0	−2.9	−13.2, 8.6	−2.6	−4.9, −0.2
25–	−1.1	−5.9, 4.0	−1.2	−3.8, 1.5	−2.7	−5.1, −0.3	−3.4	−6.3, −1.5	−2.6	−11.9, 7.7	−4.6	−7.5, 0.5
30–	−1.0	−2.7, 0.8	−0.9	−1.8, 0.1	−2.8	−4.6, −1.0	0.3	−2.3, 2.9	0.1	−1.3, 1.5	−3.2	−4.8, −1.5
35–	−1.6	−3.3, 0.1	−0.4	−1.2, 0.4	−2.4	−4.9, 0.1	−1.3	−2.0, −0.6	−0.2	−1.1, 0.6	−2.9	−3.9, −1.9
40–	0.4	−0.7, 1.5	1.9	0.9, 3.0	−1.4	−2.5, 0.2	0.3	−0.5, 1.0	1.0	0.1, 1.9	−0.8	−2.0, 0.3
45–	1.1	−2.5, 4.9	2.9	−2.1, 8.0	−1.4	−6.0, 3.4	2.2	−1.8, 6.3	3.1	1.3, 4.9	0.4	−1.0, 1.8
50–	1.0	0.5, 1.6	2.7	−1.0, 6.6	−1.7	−4.8, 1.5	1.8	0.8, 2.8	2.6	0.9, 4.4	1.0	−0.1, 2.0
55–	0.2	−0.9, 1.3	2.2	0.7, 3.6	−2.5	−3.5, −1.5	3.8	1.8, 5.9	4.6	−0.6, 10.0	2.6	1.1, 4.1
60–	0.2	−1.0, 1.4	1.5	−1.1, 4.2	−1.0	−2.6, 0.5	3.4	2.5, 4.4	3.6	2.5, 4.6	3.2	2.0, 4.4
65–	−0.3	−2.0, 1.4	1.0	−0.9, 2.9	−1.1	−2.7, 0.5	4.5	3.4, 5.6	4.5	3.1, 6.0	4.3	0.2, 8.6
70–	1.0	−2.1, 4.2	1.0	0.2, 1.7	−0.1	−0.7, 0.6	5.0	3.9, 6.1	4.8	3.5, 6.1	5.1	4.2, 6.1
75–	1.0	0.3, 1.7	1.0	0.2, 1.7	1.0	0.2, 1.8	5.1	4.2, 6.0	4.5	3.5, 5.5	5.3	4.2, 6.4
80–	2.1	1.5, 2.7	1.7	1.1, 2.4	2.4	1.8, 3.0	6.4	4.9, 8.0	5.4	4.0, 6.7	6.6	5.1, 8.2
85–	4.7	1.2, 8.3	3.6	3.0, 4.3	6.1	1.4, 11.0	7.9	6.9, 9.0	5.6	5.3, 7.8	6.1	3.1, 9.3

APC, annual percent change.

## Discussion

In this study, we observed a significant increase in diabetes mortality in China over the past three decades. The DM-CMR increased by about 170% in urban areas and 560% in rural areas between 1987 and 2019. At the same time, the DM-ASMR increased by 38.5% and 254.9%, respectively. Second, mortality due to diabetes in urban areas has been higher than in rural areas for 30 years. However, in the past decade, the gap between the two gradually narrowed due to the rapid increase of rural diabetes mortality. In addition, the mortality rate of females with diabetes is higher than that of males, but this pattern began to change slightly in 2007 in urban areas. In other words, the male mortality rate has reversed the female’s, and this change gradually became significant in 2012. Finally, China’s elderly DM mortality has shown a clear upward trend within the whole period of interest.

The overall increasing trend of diabetes mortality in China is consistent with international comparative studies on diabetes mortality patterns. Most developed countries have shown a decline in diabetes mortality in the last three decades—the United Kingdom, Germany, Nordic countries, France, and Malta (14). In contrast, a rise in diabetes mortality was recorded in most developing countries—Armenia, Bosnia and Herzegovina, Georgia, and Estonia (15). The reduction in diabetes mortality in western countries may be attributable to the availability and use of improved treatment and management, as well as the reductions in major risk factors, while the increased mortality in developing countries probably reflects lifestyle changes that accompany industrialization, including increased consumption of animal fat, obesity, and physical inactivity (16–19).

With the development of the economy and the improvement of living standards, the population’s spectrum of disease and death has undergone tremendous changes. The cost of diabetes surpasses that of other chronic diseases, posing a heavy economic burden to the current healthcare system as well as the general welfare of each family (20). The occurrence of diabetes results from many factors such as heredity and social environment. Hypertension, hyperlipidemia, overweight, obesity, smoking and drinking, and the change of social environment factors will increase the probability of diabetes (15, 21–24). Diabetes has been implicated in all-cause mortality, especially in deaths related to cardiovascular and cerebral vascular disease (25, 26). Additionally, diabetes is often associated with premature deaths from non-communicable diseases (27) as well as communicable diseases, including SARS (28), H1N1 (29), and COVID-19 (30). Among adults in China, diabetes was associated with increased mortality from a range of cardiovascular and non-cardiovascular diseases (31). The obesity epidemic caused by changes in people’s lifestyle and diet is considered to be an important reason for the aggravation of the diabetes epidemic in China, and it has been proven to be one of the critical factors leading to the onset of diabetes (32–35). Over the past few decades, China’s rapid social and economic development has led to changes in people’s lifestyles, reduced exercise time, longer sitting time, poor dietary structure, and increased life and work pressure, which has led to an increasing prevalence of obesity. According to data from the China Health and Nutrition Survey (CHNS), the obesity rate rose from 4.0% in 1993 to 10.7% in 2009 (36), and the latest national prevalence estimates for 2015–2019, based on Chinese criteria, were 34.3% for overweight and 16.4% for obesity in adults (≥18 years) (37). Dietary and healthcare interventions for lowering the prevalence of diabetes are needed in both urban and rural China. Intensive glycemic control is beneficial to the treatment of diabetic microvascular complications and can reduce mortality (38). Therefore, it is necessary to strengthen the individual and social management of diabetes.

Compared with the mortality rate in rural areas in China, the urban mortality rate is significantly higher since 1987. This may be related to higher socioeconomic status, higher living standards, better medical resources, and a higher diagnosis rate (20). However, the annual growth rate of diabetes mortality in rural residents was higher than that in urban areas in recent years, and the gap between the two gradually narrowed. Similarly, a study of the trends in diabetes mortality in China from 2003 to 2012 indicated that the diabetes mortality rate was generally higher in urban areas than in rural areas, but the gap was narrowing (39). Studies have pointed out that the level and accessibility of regional medical services will have a greater impact on diabetes mortality (40). Although the medical service and medical insurance system in rural areas of China have been established paralleling its urban part (41), their capacity is still substantially weaker than that in urban areas (42), leading to poor corresponding treatment and management of diabetes. Furthermore, socioeconomic status is also an essential factor affecting the prevalence of diabetes and its complications (43). People in rural areas have relatively low socioeconomic status, which is a potential risk factor. Accelerating urbanization could also be one reason (39), which could lead to changes in lifestyle and reduced physical activity among rural residents. In addition, studies have shown that education affects health awareness, with higher levels of education associated with higher levels of health awareness, which is related to greater access to health services and a lower risk of mortality (20). The low level of education in rural areas may lead to backward awareness of diabetes prevention and control.

Our study also found gender differences in the pattern of time trend from DM mortality. In general, whether in rural or urban areas, the mortality rate of DM among females is higher than that of males, but the pattern began to reverse in urban areas in 2007 and became significant in 2012. Studies on gender comparison of diabetes mortality in China found that the mortality rate of females was generally higher than that of males, which may be related to the influence of sex hormones or other physiological and biochemical factors in females (44). Increased social, competitive pressures may also exacerbate unhealthy lifestyles such as smoking and alcohol consumption among men, thus increasing the risk of death due to diabetes (20).

The results of this study are also supported by many relevant domestic research results. An analysis of DM mortality rate in Gusu District, Suzhou, Jiangsu province, from 2011 to 2019, showed that males had higher crude and age-standardized diabetes mortality rates than females, which may be related to the prevalence of chronic disease risk factors such as overweight, obesity, alcohol consumption, smoking, and insufficient intake of vegetables and fruits in male. And the average annual growth rate of the probability of premature death due to diabetes in males is 2.6 times that in females, suggesting that the risk of death due to diabetes in young males is higher than that in females (44). A study on the disease burden of DM in Guangzhou, China, from 2017 to 2019 shows that the loss from DM among male and female residents is 3.92 and 3.12 disability-adjusted life years (DALYs) per 1,000 population, respectively (45), and some studies attributed the gender difference in diabetes mortality to the difference in the age structure of the male and female populations (46). A gender-disaggregated analysis of DM mortality in Wujin District of Changzhou city from 2009 to 2019 showed that non-demographic factors contributed more to diabetes mortality in males, while demographic factors contributed more to diabetes mortality in females (46).

From the perspective of age-specific diabetes mortality, China’s DM mortality of the elderly population has shown a clear upward trend within the whole period of interest. Diabetes is a typical disease of the elderly. It has been identified that the age effect is a very important risk factor for diabetes mortality (39). A study on the prevalence of diabetes among residents aged 15 and above in Chongqing, China, in 2018, shows that the prevalence of diabetes increases with age (47), which leads to a corresponding increase in the death rate from DM. One of the main reasons why diabetes is more common in the elderly is that the ability to use glucose is reduced due to functional deterioration. As the aging of the Chinese population further deepens, the problem will become more prominent. Diabetes is one of the leading causes of blindness, amputation, heart disease, renal failure, and premature death in the elderly (48–52). For older adults with DM, preventing diabetic complications is the top priority. This requires comprehensive management of elderly diabetic patients, including the control of multiple risk factors such as hyperglycemia, hypertension, dyslipidemia, overweight and obesity, and hypercoagulability, and necessary drug treatment based on lifestyle intervention. At the same time, it is also necessary to set individualized control goals according to the patient’s age, disease severity, life expectancy, the severity of complications or comorbidities, etc. In addition, we should focus on strengthening the diabetes management of older women to control the rapid increase in their diabetes mortality.

### Strengths and Limitations

A strength of the present study is that the data available were of an extensively long period. Although the government has publicly published relevant data after 2003, the data before 2003 are not for the public. Relevant government departments have authorized this research to use data from earlier years for analysis. To our knowledge, this study has the most prolonged period to quantify the trends of DM mortality, which can better reflect the long-term trend of the diabetes death pattern of China. In addition, we also analyzed the heterogeneity of diabetes deaths in China between urban and rural areas and between different genders.

The potential limitations of this study include the following. First, during the study period, the classification system for coding the cause of death changed from ICD-9 to ICD-10. However, previous studies of comparability between ICD-9 and ICD-10 only observed slight differences in definitions of coding methods, which did not generate distortions in the number of deaths due to DM in essence (53). Yet China first adopted the ICD-10 international disease classification statistical standard in 2002. Some urban and rural death cause statistics stations did not submit ICD-10 death cause statistics annual reports at that time, so the mortality rate was relatively low, and consequently, the classification of some death causes was not accurate. This situation gradually improved, and the mortality rate became more accurate around 2007. The decline in overall mortality in the years after 2002 is related to this, but this does not affect the overall trend analysis and the main conclusions of this study. Second, the joinpoint regression model cannot explain the covariates or influencing factors of the outcome variables. In addition, the micro-data of DM cases are inaccessible, leading to difficulty in analysis flexibility such as calculating the overall mortality rate from urban and rural areas. Despite these limitations, this study helped to elucidate diabetes death trends in China, which still need to be clarified in analytical epidemiological studies in the future.

## Conclusions

The mortality rate of diabetes is on the rise in China. The rapid growth of the mortality rate of diabetes in rural areas leads to a shrinking urban–rural gap. Male mortality rates in urban areas have surpassed those of women. The elderly group sees a higher mortality rate of diabetes. As China’s population ages, the burden of death and disability caused by diabetes and its complications will continue to increase. More prevention and intervention policies are needed, including establishing a monitoring system for identifying early DM patients, strengthening intervention and rehabilitation for diabetic patients, and advocating a healthier lifestyle.

## Data Availability Statement

Publicly available datasets were analyzed in this study. These data can be found here: https://data.cnki.net/area/Yearbook/Single/N2020020200?z=D09.

## Author Contributions

BS and XZ conceived and designed the subject. BS and YW analyzed the data. All authors were involved in writing the paper and had final approval of the submitted and published versions.

## Conflict of Interest

The authors declare that the research was conducted in the absence of any commercial or financial relationships that could be construed as a potential conflict of interest.

## Publisher’s Note

All claims expressed in this article are solely those of the authors and do not necessarily represent those of their affiliated organizations, or those of the publisher, the editors and the reviewers. Any product that may be evaluated in this article, or claim that may be made by its manufacturer, is not guaranteed or endorsed by the publisher.

## Footnotes

1 The latest data available from: http://www.diabetesatlas.org.

## Abbreviations

WSP, World Standard Population; ICD, International Classification of Diseases; APC, annual percentage change; AAPC, average annual percentage change; CMR, crude mortality rate; ASMR, age-standardized mortality rate; IDF, International Diabetes Federation.
